# Pontine Infarction and Vertebral Artery Dissecting Aneurysm as the First Presentation of Behçet’s Disease: A Case Report

**DOI:** 10.7759/cureus.29204

**Published:** 2022-09-15

**Authors:** Kafaf S Jalali, Mohammed Ahmed Alhazzaa, Sultan Alqahtani, Mahmood Yassin Alattas

**Affiliations:** 1 Internal Medicine, Prince Mohammed Bin Abdulaziz Hospital/Saudi Commission for Health Specialties, Medina, SAU; 2 Neurology, King Fahad Hospital, Hofuf, SAU; 3 Interventional Neuroradiology, Medical Imaging Administration, King Fahad Medical City, Riyadh, SAU; 4 Rheumatology, King Salman Bin Abdulaziz Medical City, Riyadh, SAU

**Keywords:** behçet’s disease, anatomical anomaly, duplicated vertebral artery, vertebral artery dissecting aneurysm, pontine infarction

## Abstract

Behçet’s disease (BD) is a systemic disease of inflammatory origin that appears most often in the third or fourth decade of life. Behçet’s disease is hallmarked predominantly by mucocutaneous lesions and ocular involvement. Vertebral artery dissection and neurological manifestations are rare complications in Behçet’s disease. We examine the case of a medically free 33-year-old male who was admitted to the emergency department complaining of sudden-onset dizziness, vomiting, and tinnitus. Neurological examination revealed fluctuating consciousness, multiple gaze nystagmus, motor deficit in the upper and lower limbs, bilateral Babinski sign, and truncal ataxia. Magnetic resonance imaging (MRI) showed a right pontine hyperintense lesion on T2-weighted images (T2WI). A right vertebral angiogram four months after the incident showed a dissection in the mid-cervical third of an anomalous duplicated origin arm of the right vertebral artery. This case describes an uncommon form of initial presentation of Behçet’s disease via a pontine infarction triggered by a dissecting aneurysm in an anatomically rare variant of the vertebral artery.

## Introduction

Behçet’s disease (BD) is a multisystem vasculitis with a chronic, relapsing-remitting course. It is classified as a variable vessel vasculitis since it affects blood vessels of any size and type [1]. Recurrent oral mucosal and genital aphthous ulcers, and ocular lesions are key features of BD. However, other organ systems may be involved throughout the course of the disease, separated by periods ranging from a month to several years. Disease manifestations can vary greatly to include mucocutaneous, musculoskeletal, ocular, neurological, vascular, and gastrointestinal involvement [2].

BD has a global distribution, and it is known as Silk Road disease in countries such as Japan and China and spreads over to Turkey and Iran [3]. The highest prevalence is reported in Turkey, which is estimated at 421 per 100,000 population, followed by Iran, Israel, and Japan, with estimated prevalence rates of 80, 15.2, and 13.5 per 100,000 inhabitants, respectively [3]. BD has a rare presence in Central and Southern Africa, Greenland, and Australia [4]. A higher percentage of the cases in countries with low prevalence rates is seen among inhabitants whose ancestry is traceable to regions of higher prevalence [5]. In the Middle East, certain ethnic groups show a higher predisposition compared to others [6]. The reported prevalence in Qassim, Saudi Arabia, is 20 per 100,000 of the population [7]. In comparison to other Arab countries, the indigenous community in Kuwait shows a much lower prevalence of the disease [8].

The mean age of onset of BD is between the second and fourth decade of life, while pediatric-onset cases are uncommon. Moreover, the disease is exceptionally rare in people over 55 years. In the Mediterranean region, males are more commonly affected, and in Oriental countries, such as Northern European countries and the USA, the disease has a female preponderance [9]. Geographical variation is also noted in disease presentation among different groups. The diagnosis of BD is in accordance with the International Criteria for Behçet’s Disease (ICBD). It is a scoring system that includes a pathergy test. It is a phenomenon that is optional in scoring and is a nonspecific hyperreactivity response of the skin or mucous membrane to needle-induced trauma, which is characterized by pustule or papule development 24-48 hours after sterile needle prick. Over 50% of cases from the Silk Route including Turkey and Japan have a positive pathergy test, whereas it is mostly negative in cases from Northern Europe, the USA, and Australia [10].

It is widely believed that environmental and microbial risk factors such as viruses, bacteria, pollution, diet, or stress trigger an autoinflammatory response in individuals who possess certain disease susceptibility genes, such as human leukocyte antigen (HLA)-B51 or HLA-A26 [11,12]. The mechanism of damage is immune-mediated and occurs mainly via neutrophils and neutrophil-derived products [13]. BD primarily targets vascular endothelial cells causing inflammation and thrombosis. Although vasculitis is a principal pathognomonic finding in all patients, some cases of BD present predominantly with vascular manifestations, alone or in conjunction with other systemic manifestations [1]. Such cases are grouped as vasculo-Behçet’s disease. Studies have shown varied prevalence rates of vasculo-Behçet’s disease ranging from 7.7% to 43% depending on the ethnicity of the patient population [14]. Although venous involvement is more common, arteries and capillaries may also be affected. Arterial involvement is seen in 3%-5% of patients, and arterial vasculitis may present as aneurysms including false aneurysms, thrombosis, stenosis, and/or aortitis [15]. Patients with arterial involvement reported a 2.5 times higher frequency of venous involvement compared to cases without arteritis [15]. Furthermore, arterial involvement appears to be higher in males. In fact, thrombosis-related manifestations have been reported as the presenting feature in 19% of males as compared to 8% of females [14]. This report describes a unique case of Behçet’s disease involving a dissecting aneurysm in an anomalous duplicated vertebral artery origin arm accompanied by a basilar artery occlusion and subsequent infarction in the pons.

## Case presentation

A 33-year-old male with no known comorbidities and significant family history presented to the emergency department complaining of dizziness, tinnitus, and vomiting. He is a chemical engineer and married, and denies having a history of smoking or substance abuse. He reported a history of increased intensity of exercise during the two weeks prior to the incident and denied using any hormonal or steroidal supplements.

Upon further evaluation of his presenting symptoms, the dizziness was found to be acute in onset and occurred for a duration of two hours. The patient described the dizziness as a spinning (light-headedness) sensation suggestive of vertigo. The tinnitus occurred as a single, acute episode lasting for a few minutes and is present unilaterally on the right side, low-pitched in nature, and of a pulsatile pattern. Two episodes of vomiting were also described that was abrupt in onset, separated by a duration of 30 minutes. A review of his medical history and physical examination revealed a history of recurrent oral aphthous ulcers, small in size and painful in nature, that occurred once a month and lasted for a duration of 3-5 days for five years with an interval of 2-3 months. The neurological assessment revealed horizontal nystagmus without any other neurological manifestation, while initial laboratory investigations revealed no abnormalities. A computed tomography (CT) scan of the brain was done, which was normal. He was assigned a diagnosis of benign positional vertigo and discharged.

About 5-6 hours later, he was rushed back to the emergency department with impaired consciousness, slurred speech, and sudden onset of tonic postures of the upper and lower extremities that mimicked seizures and truncal ataxia. These presented as paroxysmal episodes that lasted for seconds at a time. The patient was hemodynamically stable. His temperature was 36.5°C, heart rate was 80 beats/minute, and blood pressure was 150/80 mmHg with an oxygen saturation of 99% on room air. A complete neurological examination revealed several findings. The patient showed a fluctuation in consciousness level with a score of 12 initially on the Glasgow Coma Scale (GCS). Medical Research Council muscle power assessment showed a motor deficit in the upper and lower limbs, grading of 3 on the left side and 4 on the right side, with intact sensation. The reflexes were exaggerated on both sides with bilateral Babinski sign. Cranial nerve examination showed multiple gaze-evoked nystagmuses, mild deviation of the angle of the mouth to the right, and absent gag reflex. His GCS score dropped to 6 within one hour with the onset of incontinence and an increase in the frequency of the tonic postures. He was moved to the ICU, intubated, sedated, and started on anticonvulsant therapy. Regarding laboratory investigations, C-reactive protein (CRP) elevation was noted on the fourth day of admission at 111.9 mg/L and subsided by the eighth day to 21.7 mg/L. Other tests including complete blood count, random blood glucose level, glycated hemoglobin level, serum electrolyte levels, renal and liver enzyme levels, lipid profile, and thyroid function tests revealed normal findings. Cerebrospinal fluid analysis revealed normal opening pressure at lumbar puncture, normal glucose and protein levels, and no white blood cells (WBCs) or erythrocytes. In addition, urine analysis with toxicology screening was normal. Meanwhile, electroencephalography (EEG) showed no evidence of seizure activity.

**Figure 1:**
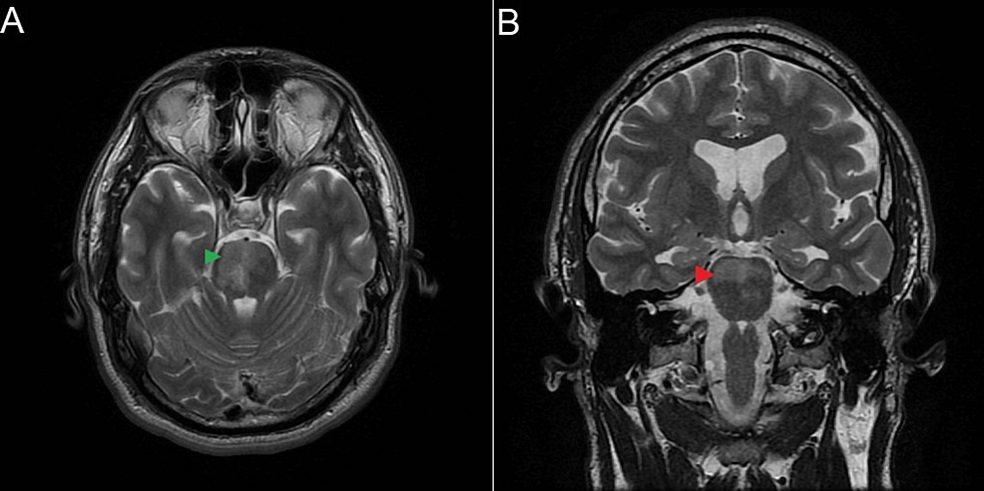
(A) Axial view brain MRI showing pontine infarction (green arrowhead). (B) Coronal view brain MRI showing pontine infarction (red arrowhead). Both images show pontine infarction. Brain MRI in axial and coronal T2WI shows high signal intensity involving the bilateral anteromedial aspects of the pons more prominent on the right side. MRI: magnetic resonance imaging; T2WI: T2-weighted image

After 40 hours of initial presentation, magnetic resonance imaging (MRI) and magnetic resonance angiography (MRA) were done and showed acute pontine infarction with radiological evidence of basilar artery occlusion suspected from a thrombus (Figure 1). Since the patient was beyond the time window for thrombectomy, treatment with aspirin 325 mg loading doses was initiated, followed by a maintenance dose regimen of aspirin 81 mg and atorvastatin 40 mg.

On hospital day 4, his CRP level rose markedly to 111 mg/L. Repeated MRI showed no new insult or hemorrhagic transformation but noted an increase in the size of the peri-infarction edema for which he was started on a high-dose methylprednisolone regimen of 1 g/day for five days. CT angiography of the brain and neck was done to rule out any evidence of dissection, and it was unremarkable. Electrocardiography (ECG), echocardiography, 48-hour Holter monitoring, and carotid ultrasonography found no abnormalities. Rheumatologic tests for antinuclear antibody (ANA), anti-double-stranded DNA antibody, antineutrophil cytoplasmic antibodies, factor V Leiden, antithrombin III, bone GLA protein, immunoglobin A, immunoglobin G, immunoglobin M, complement C3, complement C4, homocysteine, and cryoglobulin were unremarkable; however, lupus anticoagulant test was weakly positive (Table 1). The patient also tested negative for human immunodeficiency virus, hepatitis C virus, hepatitis B virus, and syphilis infections.

**Table 1:** Vasculitis and thrombophilia investigations. Anti-dsDNA: anti-double-stranded DNA; ELISA: enzyme-linked immunosorbent assay; ANA-IFA: antinuclear antibodies-immunofluorescence assay; P-ANCA MPO: perinuclear anti-neutrophil cytoplasmic antibodies; C-ANCA PR3: antineutrophil cytoplasmic autoantibody, cytoplasmic; LA: lupus anticoagulant

Examination name	Value	Reference range
Homocysteine	9.31	7.40-19.80 umol/L
Anti-dsDNA (ELISA)	51.01	0-200 -ve
ANA-IFA	Negative	Negative
P-ANCA MPO	8.13	≤20 -ve
C-ANCA PR3	7.61	Unit
Rheumatoid factor	<10	<15 IU/ML -ve
LA	Weakly present	
Functional protein C	82	70%-140%
LA1/LA2 ratio	1.4	
LA1	53	26-42 seconds
LA2	39	Seconds
Bermuda grass pollen IgA	1.76	<20, negative
Bermuda grass pollen IgG	0.55	<20, negative
Bermuda grass pollen IgM	1.29	<20, negative
Anticardiolipin antibodies, IgA	3.83	APL unit(s)
Anticardiolipin antibodies, IgG	9.15	GPL unit(s)
Anticardiolipin antibodies, IgM	6.5	MPL unit(s)
Human leukocyte antigen B51	Negative	

On hospital day 8, the patient’s GCS score rose to 10-11. He was opening his eyes spontaneously and responding to verbal commands. He was also able to maintain an oxygen saturation of 95%-97% on room air and was successfully extubated without any complications. The following day, a repeated muscle power assessment revealed a grade 3 right limb motor deficit and a grade 0 left limb deficit. Complete aphasia was also noted. Swallowing assessment revealed dysphagia and evidence of aspiration, which necessitated nasogastric intubation. All inflammatory markers normalized during the subsequent hospital stay, and he was continued on aspirin and atorvastatin.

**Figure 2:**
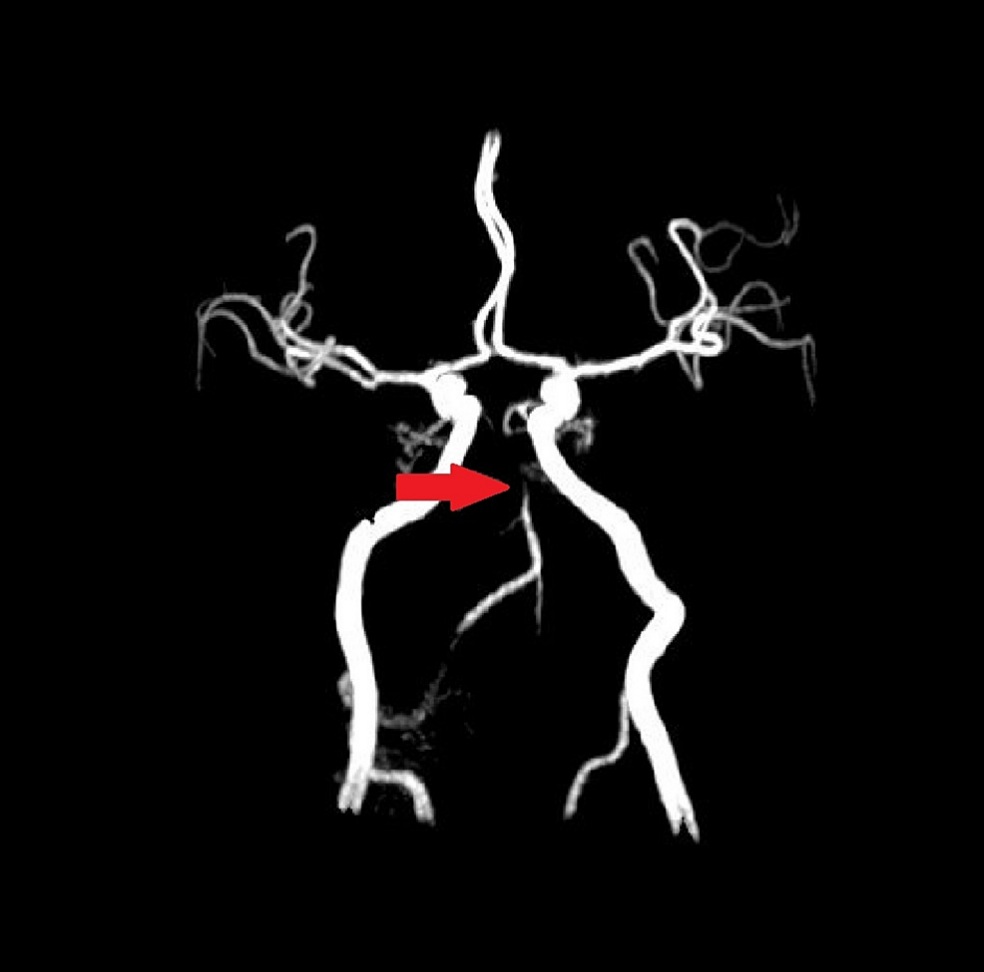
Magnetic resonance angiography showing severe distal vertebral artery stenosis (red arrow).

On hospital day 10, the patient was transferred to a higher facility for further investigations and a comprehensive rehabilitation program. Transesophageal echocardiography showed no evidence of thrombus or shunting. Cerebral angiography revealed distal basilar occlusion with minimal, partial recanalization with the retrograde filling of the basilar apex through bilateral posterior communicating arteries. Lack of atherosclerotic disease findings in the intracranial and extracranial vasculature imaging led to a diagnosis of distal basilar occlusion most likely resulting from an embolic thrombus (Figure 2).

There was no evidence of dissection, but anticoagulant therapy was initiated. The patient was administered a heparin infusion of 18 units/kg/hour and was later switched to warfarin after a neurological examination. A maintenance anticoagulant regimen with a target international normalized ratio (INR) of 2-3 was recommended to prevent any further embolization from a hidden dissection since he had a history of extensive exercise immediately prior to the stroke.

**Figure 3:**
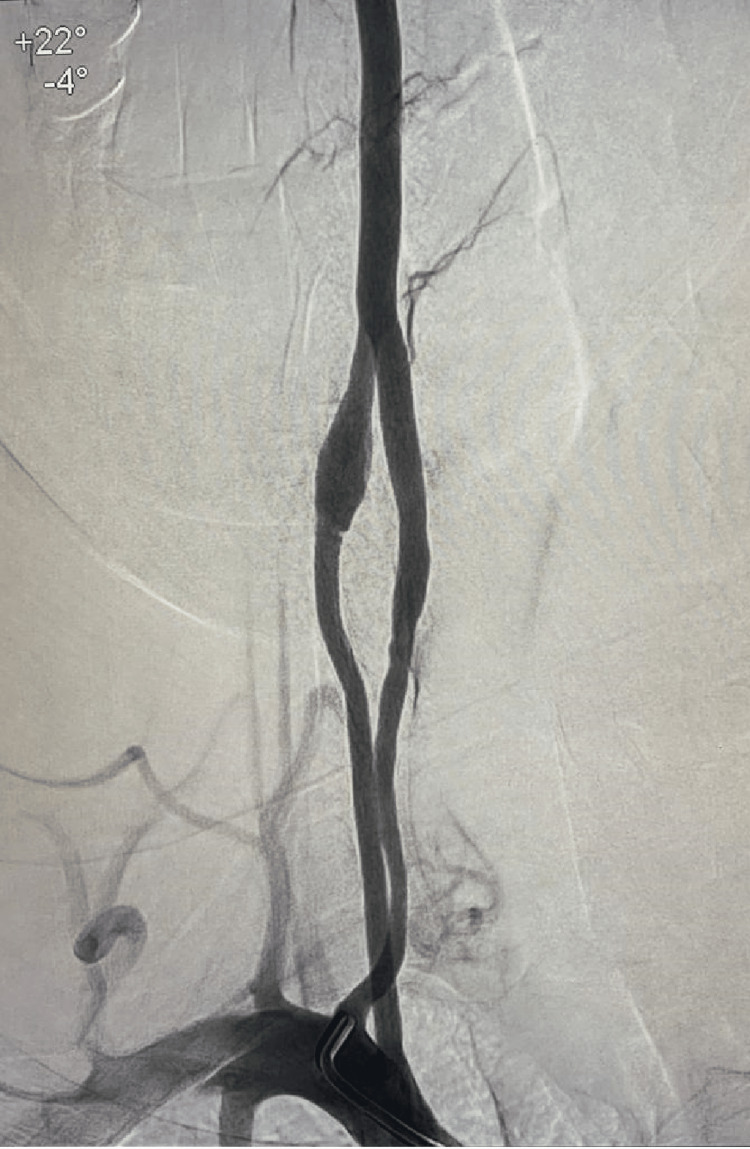
Cerebral vessel angiography showing significant anomaly involving the origin of the right vertebral artery, which is bifid and duplicated from its origin to the mid-cervical third (C3) where it shows a fusiform dissecting aneurysm in one of the origin arms.

After completing three weeks in the acute stroke unit, he attended a five-week comprehensive inpatient rehabilitation program in multiple dimensions (physiotherapy, occupational therapy, vocational therapy, speech, swallowing, and sexual rehabilitation). Following this, he completed one week of outpatient rehabilitation with good ongoing recovery. Four months after the initial presentation, cerebral angiography was repeated, which showed significant recanalization of the severely occluded distal part of the basilar artery segment with just minimal residual narrowing at the distal tip and posterior P1 segments of cerebral arteries bilaterally. A significant anomaly was noted involving the origin of the right vertebral artery, which was bifid and duplicated from its origin to the mid-cervical third where it showed a fusiform dissecting aneurysm in one of the right vertebral origin arms, an observation that was not noted in the previous examination. The patient underwent conventional cerebral angiography and showed a vertebral artery dissecting aneurysm (Figure 3). Stenting was considered, but since the patient had no recurrent stroke in the four months following the incident, a conservative management approach was adopted, which included repeated cerebral angiography every six months to follow the size of the dissecting aneurysm and plan future interventions accordingly.

The patient was seen by a rheumatologist for the suspicion of BD considering the history of recurrent mouth aphthous ulcers, along with the pontine infarction and vertebral artery dissecting aneurysm. No other manifestations of BD were noted. A pathergy test and HLA-B51 typing were performed and reported negative. Investigations for thrombophilia, including APS findings, were negative too, and continuously normal CRP and erythrocyte sedimentation rate (ESR) levels and WBC count were reported. A comprehensive ophthalmic examination reported normal findings. Chest and abdominal CT with IV contrast revealed normal medium and large vessels. Warfarin was stopped, and a regimen comprising aspirin 81 mg once daily, azathioprine dose of 150 mg once daily, and colchicine dose of 0.5 mg twice daily was initiated. This was considered for the conservative medical management of the vertebral artery dissecting aneurysm and clinically suspected Behçet’s disease.

Cerebral angiography was repeated six months later and showed no changes. It redemonstrated the duplicated right vertebral artery with dissecting fusiform aneurysm along the distal aspect of the proximal right vertebral artery, which had an extraforaminal course and joined the other vertebral artery at the upper border of the C5 vertebral body. The nondominant left vertebral artery originated directly from the aortic arch and primarily ended as the posterior inferior cerebellar artery. A three-year follow-up of the patient showed significant improvement in his functional status. There was no development of any other BD manifestations or recurrence of any vascular insult. Serial ophthalmology examination reported normal results.

## Discussion

Underlying vasculopathies such as BD increase the risk of cerebrovascular events [16]. A dissecting aneurysm is one such form of presentation. A dissecting aneurysm results from a longitudinal tear of the vessel intima with subsequent spread of intramural hemorrhage into the subadventitial plane, causing a saccular or fusiform expansion of the artery [17]. Under rare circumstances, these events occur in the vertebral artery and precipitate hemorrhagic or thrombotic cerebrovascular incidents. Disrupted blood flow and impaired oxygen supply from such events frequently manifest as ischemic stroke. Subsequently, ischemic injury initiates tissue necrosis in the brain, leading to infarction. In the case of basilar artery occlusion caused by vertebral artery dissecting aneurysms, brain stem involvement and corresponding neurological findings are observed clinically. These findings may vary depending on the extent of blood flow compromise [18].

This case demonstrates the combination of two atypical clinical features. Firstly, the patient presents with neurological involvement due to brain stem involvement and ischemic events due to a dissecting aneurysm, both of which are relatively uncommon signs of the first presentation of BD [19]. Secondly, the patient shows duplication of the right vertebral artery, an anomalous vascular variant [20]. Interestingly, the vertebral artery dissecting aneurysm occurs in one of the origin arms of the duplicated right vertebral artery. Younes et al. reported a case of a 44-year-old male in whom the diagnosis of BD was confirmed on the basis of recurrent oral aphthous ulcers and a positive human leukocyte antigen (HLA) B51 test result [21]. Multiple artery aneurysms, subacute white matter hypersignals in the left capsule lenticular area, and an old ischemic lesion in the right capsulo-lenticular region were all visible on MRI. The patient had immunosuppressive treatment and systemic high-dose corticosteroids for two months. The author further stated that approximately 3% of patients with BD present with neurological involvement as a first sign, making it a rare presentation. In addition, the occurrence of ischemic events and aneurysms is quite limited [21]. Recurrent oral aphthous ulcers were also reported in our case; however, the HLA-B51 test was negative in our patient. Caso et al. reported a case of a 43-year-old male whose duplex examination revealed blockage of the left vertebral artery, which was supported by magnetic resonance angiography and digital angiography with usual symptoms of vertebral dissection. During the hospital stay, arthralgias with elevated inflammatory markers and oral aphthous ulcers with high fever were noted. There were no typical eye abnormalities and no vaginal ulcers, and the pathergy test was negative [22]. Elevated inflammatory markers were also observed in our patient, but similarly, the pathergy test was negative.

Although our patient reported negative for HLA-B51, studies have found HLA-B51 as an indicator of poor prognosis, specifically, an early disease progression, ocular lesions, and vascular involvement [23]. Interestingly, the relative risk of HLA-B51-carrying individuals to develop the disease does not correlate with the global geographic distribution of the allele. It is higher in a small geographic belt, which overlaps the regions along the Silk Road, including the Middle East [23]. More recent evidence indicates that rather than being directly involved in the etiology, HLA-B51 is closely linked to other disease-related gene or genes [24].

Vasculitis is the main underlying pathological feature of Behçet’s disease. The mechanism of tissue damage is mainly mediated via neutrophilic vasculitis where hyperactivated neutrophils contribute to altered fibrinogen function and thrombosis formation. Arteries are affected much less commonly than veins at 1%-6%, as opposed to about 70% [25]. Arterial lesions commonly occur as aneurysms, pseudoaneurysms, or obstructions linked with an aneurysm. Ischemic events due to BD are scarce but life-threatening in nature. No definitive biomarker, histopathological feature, or laboratory test exists for establishing a diagnosis of BD. Therefore, BD remains a clinical diagnosis determined by its disease manifestations. According to the International Criteria for Behçet’s Disease (ICBD), the proposed diagnostic criteria are distributed according to a point system. Oral aphthosis is given 2 points, genital aphthosis 2 points, ocular lesions 2 points, skin lesions 1 point, and vascular manifestations 1 point. An aggregation score of 4 or more indicates a diagnosis of BD. If a skin pathergy test is done and the result is positive, an extra 1 point is assigned [26]. However, the primary scoring system does not include it as a requirement. For the proposed criteria, ICBD defines vascular manifestations as arterial thrombosis, large vein thrombosis, phlebitis, and superficial phlebitis (Table 2) [27].

**Table 2:** International Criteria for Behçet’s Disease scoring system. ^a^A pathergy test is optional. Where a pathergy test is conducted, 1 extra point may be added for a positive result. Kiafar et al. [27]

Signs/symptoms	Points
Ocular lesions	2
Genital aphthosis	2
Oral aphthosis	2
Skin lesions	1
Neurological manifestations	1
Vascular manifestations	1
Positive pathergy test^a^	1

Several cluster analyses and association studies have found significant associations between particular disease manifestations, reported as BD phenotypes [28]. The three major phenotypes identified are mucocutaneous and articular phenotype, parenchymal neurological and ocular phenotype, and peripheral vascular and extra-parenchymal neurological phenotype. In this case, the patient’s features overlapped with the third phenotype.

Neurological presentation in BD may differ widely and is reported to have a variable presence between 2.2% and 50% [29]. Based on the disease phenotype, it may originate from primary central nervous system involvement or secondarily from vascular involvement, as seen in this case. The former category of involvement is known as the parenchymal pattern; it is more frequent and is related to an inflammatory, meningoencephalitic process. The latter category of neurological involvement is known as the extra-parenchymal pattern, where it leads to a secondary central nervous system dysfunction due to the involvement of major vessels. On rare occasions, both types of presentations can simultaneously occur, leading to a mixed pattern of presentation.

Our case fulfilled the diagnostic criteria for ICBD and presented with neurological manifestations arising from brain stem involvement in the form of pontine infarction, which was precipitated by a basilar artery occlusion suspected of resulting from the dissecting aneurysm in the right vertebral artery. A significant anomalous finding of a duplicated right vertebral artery was reported, which contained the dissecting aneurysm responsible for the acute illness onset. The vertebral artery dissecting aneurysm was located in the proximal origin arm of the vertebral artery. There are only two reports in the scientific literature of vertebral artery dissecting aneurysm occurrences in the duplicated vertebral arteries [30,31]. It is suspected that these rare anatomical variants may predispose to vertebral artery dissecting aneurysms due to one or more factors such as local microvascular defects or major hemodynamic changes. Therefore, although the duplication of the origin of the vertebral artery has no clinical implications in most cases, a preexisting inflammatory arterial disease such as BD can be considered as a risk factor for the development of an arterial dissecting aneurysm.

Regarding treatment, Shahien and Bowirrat reported in their study that prednisone was administered orally to 13 of the 14 patients who got high-dosage steroids, while 1,000 mg/day of methylprednisolone was administered intravenously to one patient, with slow tapering of the dose [32]. A mixture of steroids and cytotoxic drugs was administered to four patients. By the end of the therapy term, 87.5% of patients had seen noticeable relief. He further concluded that early, intensive treatment with cytotoxic drugs and corticosteroids may improve patients’ prognosis and lessen or stabilize the detrimental effects of neurological involvement [32]. Literature supports the role of corticosteroids and cytotoxic drugs in BD patients having neurological and vascular involvement. Our patient was also successfully treated with azathioprine and methylprednisolone.

The limitations of the report include the lack of typing for other HLA and non-HLA genes, which would have allowed further insight into the pathogenesis and prognosis of the disease. The gender and age of the patient may have contributed to the BD since the disease is more dominant among males and at younger ages, and the presence of vasculitis may have led to the occurrence of the cerebrovascular event.

## Conclusions

Vertebral artery dissecting aneurysm is an extremely rare but nevertheless severe complication of BD. The same treatment strategy employed for managing vasculitis in BD and in cases of endovascular embolization should be used to manage dissecting aneurysms related to BD. The presence of anomalous anatomical variants in rare chronic systemic diseases makes diagnosis and management of such cases more challenging. The literature is limited to case reports, and the incidence of such lesions is not known. With the increased use of noninvasive vascular imaging in the future, it may be possible to detect these types of lesions more frequently in susceptible patients.
